# Behavioral trajectories of aging prairie voles (*Microtus ochrogaster*): Adapting behavior to social context wanes with advanced age

**DOI:** 10.1371/journal.pone.0276897

**Published:** 2022-11-15

**Authors:** Jeanne M. Powell, Madison M. Garvin, Nicholas S. Lee, Aubrey M. Kelly

**Affiliations:** Department of Psychology, Emory University, Atlanta, Georgia, United States of America; Oregon Health and Science University, UNITED STATES

## Abstract

Several studies using mice have examined the effects of aging on cognitive tasks, as well as sensory and motor functions. However, few studies have examined the influence of aging on social behavior. Prairie voles (*Microtus ochrogaster*) are a socially monogamous and biparental rodent that live in small family groups and are now among the most popular rodent models for studies examining social behavior. Although the social behavioral trajectories of early-life development in prairie voles have been well-studied, how social behavior may change throughout adulthood remains unknown. Here we examined behavior in virgin male and female prairie voles in four different age groups: postnatal day (PND) 60–80, 140–160, 220–240, and 300–320. All animals underwent testing in a novel object task, a dominance test, a resident-intruder test, and several iterations of social approach and social interaction tests with varying types of social stimuli (i.e., novel same-sex conspecific, novel opposite-sex conspecific, familiar same-sex sibling/cagemate, small group of novel same-sex conspecifics). We found that age influenced neophobia and dominance, but not social approach behavior. Further, we found that young adult, but not older adult, prairie voles adapt prosocial and aggressive behavior relative to social context, and that selective aggression occurs in relation to age even in the absence of a pair bond. Our results suggest that prairie voles calibrate social phenotype in a context-dependent manner in young adulthood and stop adjusting behavior to social context in advanced age, demonstrating that social behavior is plastic not only throughout early development, but also well into adulthood. Together, this study provides insight into age-related changes in social behavior in prairie voles and shows that prairie voles may be a viable model for studying the cognitive and physiological benefits of social relationships and social engagement in advanced age.

## Introduction

Aging has become a difficult public health challenge facing most societies given the increasingly high population of older adults. Indeed, several initiatives are being considered and developed to facilitate healthy aging [1–3]. To understand factors that influence healthy aging, it is crucial that we examine natural behavioral, cognitive, and physiological trajectories associated with advanced age. Although studies in humans are highly beneficial, using non-human animal models can provide insight about aging on more rapid timescales and in tightly controlled experimental environments. While there are several studies in mice that have examined the impact of age on sensory and motor function, as well as performance in cognitive tasks, few studies have examined the influence of age on social behavior. Here we characterize behavioral trajectories of social behavior in the prairie vole (*Microtus ochrogaster*), which has emerged as a model organism for studying sociality and relevant underlying mechanisms [4–6].

Prairie voles are a small arvicoline rodent native to prairie grasslands of central and eastern North America [7, 8]. They are socially monogamous and live in extended family groups. Adult males and females form pair bonds characterized by affiliative and pair-maintenance behaviors including huddling, allogrooming, mate guarding, and mate preference [9, 10]. Pair bonded individuals also exhibit biparental care, in which both mothers and fathers engage in nest construction, food caching, grooming, pup retrievals, and brooding [11]. Additionally, non-parent family group members (i.e., typically offspring) exhibit alloparental care and readily retrieve, groom, and brood young pups [12].

Given their capacity for pair bonding, parental care, and alloparental care, prairie voles have emerged as a model organism for studying social behavior and relevant underlying neural mechanisms [5, 13]. Research using prairie voles has enabled scientists to uncover neural circuitry of monogamous bonds [14], develop a more socially-translational model for studying alcoholism and addiction [6], elucidate mechanisms underlying socially mediated stress buffering [15], examine the influence of paternal deprivation [16] and natural variation in parental care [17] on development, and understand the behavioral and physiological consequences of social isolation [18], cesarian delivery [19], and early-life exposure to anti-depressants [20]. Because prairie voles can be readily bred in the lab, many studies take a developmental approach and examine brain and behavior across the lifespan. Indeed, studies range from studying neonatal pups and weanlings to adolescents and adults [21–26].

Several studies have examined developmental trajectories of social behavior in prairie voles. For example, males reared by single mothers and subsequently weaned into social isolation exhibit greater affiliative behavior as juveniles and are more likely to form a partner preference as adults compared to males raised by a mother and a father and weaned with same-sex siblings [25]. When contrasting female juveniles (postnatal day (PND) 19–20) with female adult (PND60-90) prairie voles, juvenile behavior is characterized by less anxiety-like behavior, greater affiliation toward same-sex conspecifics, and positive interactions with pups [27]. Similarly, male juveniles (PND15) are more prosocial with age-matched, same-sex conspecifics than males that are PND30, PND45, and PND60; aggression toward same-sex conspecifics and social avoidance is also greater in PND30-60 males compared to weanling aged juveniles (i.e., PND15) [28]. Together, these studies show that prairie voles are more prosocial when they are younger and shift toward exhibiting a more aggressive phenotype as they reach adulthood. These shifts in behavior across early development correspond with major life history events and reflect the transition from a period of allopatric cohabitation with siblings to a period of time when voles disperse and prepare to establish territories and compete for mates.

Although prairie vole early life development is well-studied, there remains a need to examine whether behavior continues to change with advanced age. To our knowledge, only one previous study has examined the influence of advanced age on prairie vole behavior. In this study, Kenkel et al. found that while male prairie voles aged PND115-1046 all retain the ability to form new pairbonds and exhibit a significant partner preference, PND640-1036 males spend less time in contact with an opposite-sex stranger. Additionally, the eldest group (PND500-1036) also showed less anxiety-like behavior in an open field test [29]. Studying advanced aging will not only provide insight into specific behaviors that may exhibit more plasticity than others throughout the lifespan but, given the popularity of using voles as a model organism for social neuroscience, there is also utility in identifying an age window in which we can use voles as subjects before expecting to have natural behavioral changes associated with age serving as a confound. While, somewhat surprisingly, the majority of studies using prairie voles do not report the age of adult subjects used, many studies simply state that adult subjects were “at least 60 days of age.” In studies where the age is identified in the paper, common age ranges include PND60-90, PND70-120, PND60-180, and PND60-200 [30–35]. Of the common adult age ranges reported in studies, we know little about whether social behavior in prairie voles undergoes changes associated with aging.

To address this gap in the literature, here we examined changes in social and anxiety-like behavior in prairie voles ranging from PND60 to PND320. PND60 indicates the age in which prairie voles are considered adults; sexual maturation occurs by PND55 in males and by PND45 in females [36, 37]. Although prairie voles can live to around 3 years of age in the lab (pers. obs.), field studies suggest an average lifespan of less than 1 year [38]. In the present study, all subjects underwent testing in a novel object task, a dominance test, a resident-intruder test, and several iterations of social approach and social interaction tests with varying types of social stimuli. Because physical health in any animal deteriorates with age, we hypothesized that aggression would decrease with age given that engaging in aggressive acts is energetically costly. Further, we hypothesized that neophobic behavior would be lower at young adult ages, potentially serving to facilitate a phenotype conducive to exploring novel environments to find territories and/or mates.

## Materials and methods

### Animals

All prairie voles used in this study were bred in the Kelly Lab at Emory University. Our colony is derived from breeders (offspring of wild caught individuals in Illinois) acquired from the lab of Dr. Alexander Ophir at Cornell University. All subjects were weaned from their parents on post-natal day (PND) 21 and housed in same-sex sibling pairs with access to *ad libitum* food (Lab Rabbit Diet HF #5326, LabDiet) and water in standard rodent polycarbonate cages (29cm L *X* 18cm W *X* 13cm H) lined with sanichip bedding. Before behavioral testing, each animal was ear tagged with a unique ID number. The colony room was maintained at an ambient temperature of 24°C +/- 1°C and kept on a 14 h:10 h light/dark cycle. All procedures related to the use of animals in this study followed ASM guidelines [39] and were approved by the Emory University Institutional Animal Care and Use Committee (PROTO201900094).

Male and female same-sex sibling pairs were semi-randomly assigned to each age group (PND 60–80, 140–160, 220–240, and 300–320) to ensure mixed genetic heritage within each group. Age groups were chosen to represent the span of ages that researchers commonly use as subjects in their studies. All subjects were sexually naïve. Behavioral data from 5 animals were not included in analyses due to corrupted or lost video files. Sample sizes for data analysis: PND60-80, n = 11 males and 13 females; PND140-160, n = 13 males and 10 females; PND220-240, n = 9 males and 12 females; PND300-320, n = 9 males and 10 females. Total n = 87.

### Experimental design

In total, each subject participated in 12 assays; the subject was the focal animal in 10 of the assays and the stimulus animal for their same-sex sibling/cagemate in the remaining 2 assays (i.e., social approach and social interaction with a familiar, same-sex conspecific). All testing took place while the subjects were aged within their assigned age window. The testing order was randomly generated for each prairie vole using a random number generator, and each subject participated in a maximum of 3 assays per day with roughly an hour break between each test. Running up to 3 assays per day was a requirement due to campus restrictions related to the Covid-19 pandemic to reduce the number of personnel on campus. Notably, being run through 3 tests in a day may have served as a mild stressor for subjects. Because the order of behavioral tests was randomized, if stress influenced behavior, effects would be present across all tests. All behavioral tests were video recorded using Sony Handycam HDR-CX405 1080p Camcorders (Sony, USA) for subsequent scoring using Behavioral Observation Research Interactive Software (BORIS; Friard & Gamba [40]). Subjects were not tested at multiple ages because brains were collected at the end of each age window for a different experiment not discussed here.

### Novel object investigation

To assess neophobia, subjects were acclimated to a clean rodent cage with sanichip bedding for 20 min. After acclimation, a sterilized novel object was placed in the cage. The novel object for all subjects was a clear, purple-capped 50 mL centrifuge tube filled with crumpled cyan sticky notes. Subjects were given 10 min to interact with the object. The first 7 min of the assay was scored to quantify the latency to approach, time spent near, and time spent investigating the object. See Table 1 for ethogram.

**Table 1:** Ethograms for behavioral assays.

Dominance Tube Test	
**Win**	Subject animal remains in tube and the stimulus animal has fully exited the tube with all four paws on the ground.
**Lose**	Subject animal fully exits the tube with all four paws on the ground while the stimulus animal remains in the tube.
**Tie**	Neither animal fully exits tube within 3 minutes.
Novel Object Investigation	
**Latency to approach**	Time at which subject’s nose is first within 1 cm of the object.
**Time near object** *	Subject is within one body length of the object but does not need to be oriented towards or actively investigating the object.
**Time investigating object** *	Subject’s nose is within 1 cm of the object.
Social Approach Test	
**Latency to approach**	Time at which subject is first within one body length to the barrier.
**Time near stimulus** *	Subject is within one body length of the barrier but does not need to be oriented towards or actively investigating the subject.
**Time investigating stimulus** *	Subject’s nose is within 1 cm of the barrier.
Resident Intruder & Social Interaction Tests	
*Prosocial Behavior*	
**Allogrooming**	Subject grooms the stimulus animal.
**Huddling**	Subject and stimulus animals are either touching flanks or laying on top of each other.
**Positive investigation**	Subject sniffs or investigates stimulus animal without apparent aggression.
**Positive side-by-side contact**	Subject and stimulus animal are passively touching sides without apparent aggression.
*Aggressive Behavior*	
**Aggressive side-by-side contact**	Subject and stimulus animals are touching flanks in an aggressive manner, sometimes between aggression bouts.
**Biting**	Subject bites the stimulus animal.
**Chasing**	Subject is aggressively chasing the stimulus animal and is the initiator for the entire event. Does not include non-aggressive following.
**Lunging/Attacking/Rolling**	Subject lunges at, swipes paws at, or rolls around with stimulus vole in aggression.
**Pinning**	Subject pins stimulus down.
**Rearing**	Subject is rearing up on hind paws in either offense or defense.
*Nonsocial Behavior*	
**Autogrooming**	Subject grooms itself while not in contact with the stimulus animal.

* denotes that the behavioral state can co-occur with other states within an assay. Otherwise, all behavioral states are mutually exclusive.

### Dominance tube test

Subject and stimulus animals were simultaneously placed in opposing ends of a clear, plexiglass tube (30.25cm length with an 8.25cm diameter for smaller voles and 10cm diameter for larger voles) and were video recorded until the more dominant animal pushed the subordinate animal out of the tube. The dominant animal was scored as the winner and the subordinate animal as the loser. If an animal backed out of their own accord, we presumed this indicated subordinance and the trial was marked as a loss. If at the end of a 3-min period neither animal had exited the tube, the trial was ended and considered a tie. Stimulus animals were novel, same-sex individuals weighing within 5 g of the subject animal. This assay was repeated for 3 trials. The side of tube entry was counterbalanced to ensure handling did not impact performance.

### Modified resident intruder test

Because we tested both animals in a sibling pair that shared a homecage, we used a modified resident-intruder paradigm such that subjects were not tested in their homecage. Instead, subjects were placed in a clean rodent cage lined with sanichip bedding and were able to “establish residence” in a longer acclimation period of 20 min. Stimulus animals were age- and weight-matched novel, same-sex conspecifics. Stimuli were fitted with a vibrant zip-tie collar to distinguish them from the subject animal and were given 20 min in their homecage to acclimate to the collar before testing. After the subject acclimation period, the stimulus animal was placed into the subject’s cage for 6 min. The first 5 min of each interaction was scored to measure prosocial, aggressive, and non-social behaviors. See Table 1 for ethogram. Because distinct types of behaviors can occur infrequently, we combined all prosocial behaviors into a single prosocial behavior measurement and all aggressive behavior into a single measurement of aggression.

### Social approach test

The social approach assay allows subject animals to approach and investigate stimulus animals without directly interacting with them by separating the two individuals with a perforated, plexiglass barrier. To do this, subjects and stimulus animals were placed in separate clear, plexiglass chambers (subject chamber, 45cm L *X* 13cm W *X* 13cm H; stimulus chamber, 13cm L *X* 13cm W *X* 13cm H) and allowed to acclimate to their surroundings for 10 min. At the end of the acclimation period, the subject was placed under a beaker at the unperforated side of their chamber furthest away from the stimulus chamber; the perforated end of the stimulus chamber was then placed adjacent to the perforated wall of the subject chamber. The subject was released from under the beaker and behavior was recorded for 8 min. The first 5 min of the test was scored for the latency to approach, times spent investigating, and time spent near the stimulus animal. This assay was run 4 times for each subject, each time using a different type of stimulus animal. Stimulus animal types included a novel same-sex conspecific, a novel opposite-sex conspecific, a familiar same-sex sibling/cagemate, and a novel same-sex group of 3 stimulus animals (stimuli were co-housed siblings). See Table 1 for ethogram.

### Social interaction test

The social interaction assay allows subjects to freely interact with stimulus animals. 20 min prior to the start of testing, stimulus animals were zip-tied for identification purposes and returned to their homecage. To create a neutral environment in which neither animal was a resident of the territory, subject and stimulus animals were concurrently placed in a clean rodent cage with clean sanichip bedding. Behavior was video recorded for 11 min, and videos were scored for prosocial, aggressive, and non-social behaviors (see Table 1). This assay was run 3 times for each subject, each time using a different type of stimulus animal. Stimulus animal types included a novel same-sex conspecific, a novel opposite-sex conspecific, and a familiar same-sex sibling/cagemate. Because distinct types of behaviors can occur infrequently, we combined all prosocial behaviors into a single prosocial behavior measurement and all aggressive behavior into a single measurement of aggression.

### Statistics

Behavioral data were analyzed using General Linear Models (GLM), repeated measures GLMs (RM GLM), Linear Mixed Models (LMM), and Friedman’s test. All posthoc pairwise comparisons were adjusted using the Sidak correction. All data were analyzed using SPSS 28 (IBM Analytics, USA) and graphs were made using Prism 8 (GraphPad, USA).

## Results

Here we sought to determine whether distinct aspects of behavior change with advanced age in prairie voles. Additionally, we examined effects of sex on behavior and how sex may interact with age to influence behavior trajectories throughout adulthood. Male and female prairie voles were tested in a novel object investigation task to assess neophobia, a dominance test, a resident-intruder test, and social approach and social interaction tests in varying social contexts.

### Novel object investigation

**Fig 1:**
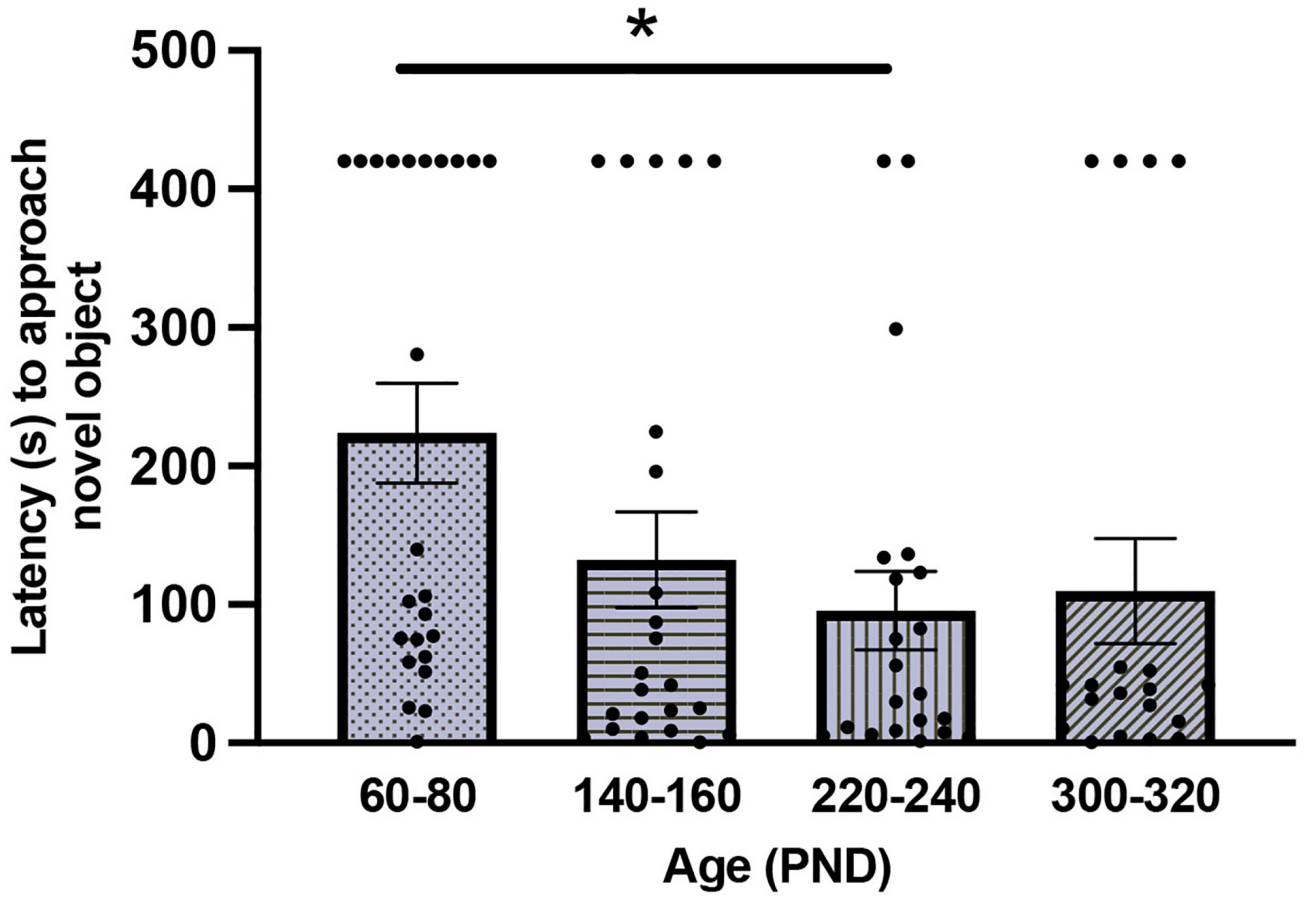
Mean ± SEM time (seconds, s) to approach a novel object for each age group. Voles between PND60-80 exhibited a greater latency to approach the object compared to voles aged PND220-240. Dots represent individual data points. * indicate statistical significance.

When examining the latency to approach a novel object, a GLM with Sex and Age as fixed factors found a main effect of Age (F_(3,86)_ = 2.81; *P* = 0.05; Fig 1). Sidak-corrected posthoc analyses revealed a significant difference between PND60-80 and 220–240 (*P* = 0.05), with PND60-80 animals taking longer to approach the novel object (all other posthoc comparisons *P* > 0.13). We observed no effect of Age on time spent near or time spent investigating the novel object (all *P* > 0.14). Additionally, we found no effects of Sex (all *P* > 0.36) and no significant interaction between Age and Sex (all *P* > 0.22) for the latency to approach, time spent near, or time spent investigating the novel object.

### Dominance test

**Fig 2:**
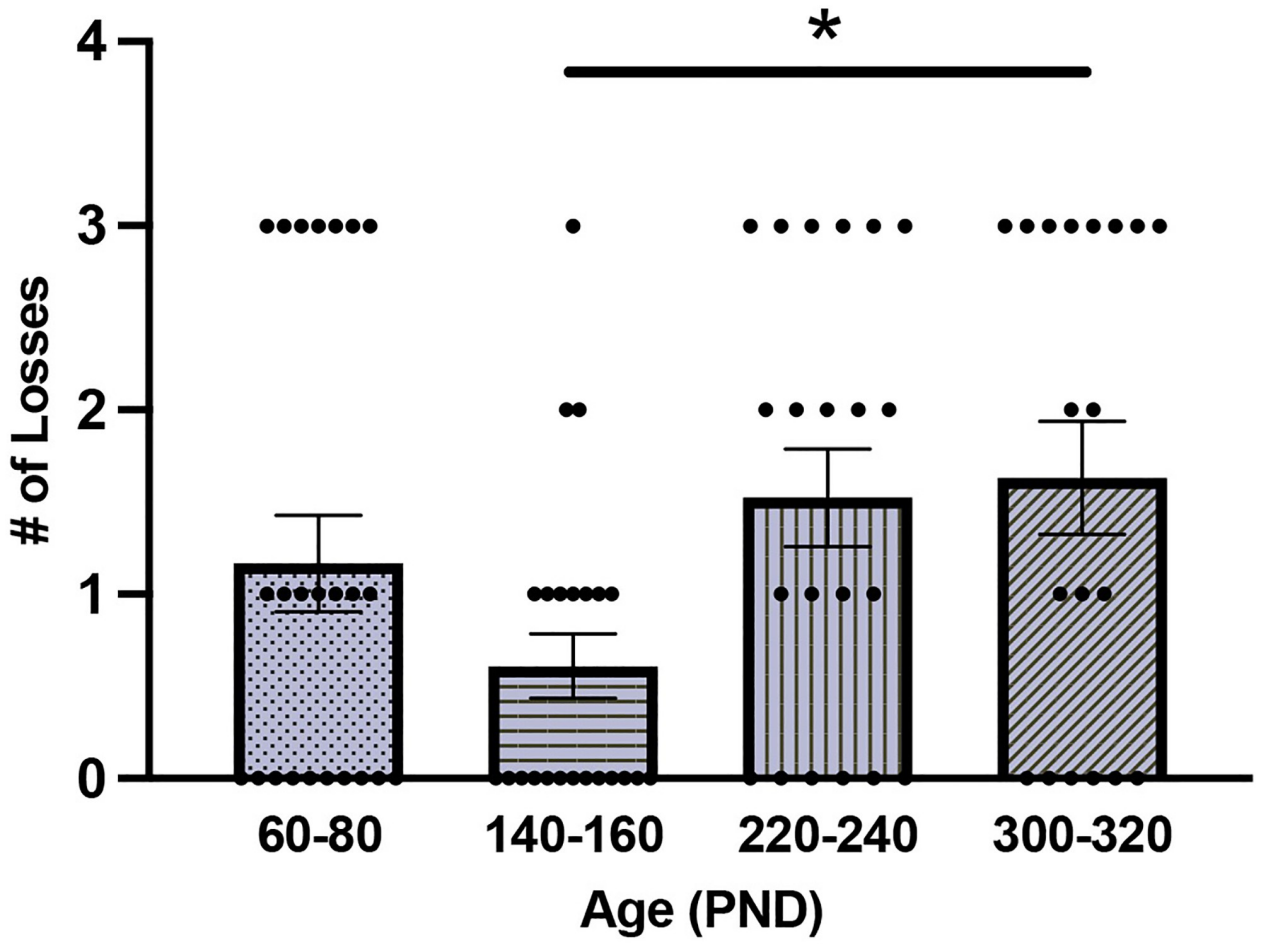
Mean ± SEM the number of losses in the dominance test for each age group. Voles between PND140-160 lost fewer trials compared to voles aged PND300-320. Dots represent individual data points. * indicate statistical significance.

For the dominance test, a GLM with Sex and Age as fixed factors found a main effect of Age on the number of losses out of 3 trials (F_(3,86)_ = 3.31; *P* = 0.02; Fig 2). Sidak-corrected posthoc analyses revealed a significant difference in the number of losses only between PND140-160 and PND300-230 (*P* = 0.04), with the younger age group losing less compared to the eldest age group (all other posthoc comparisons *P* > 0.07). However, we found no main effect of Age on the number of wins or ties (all *P* > 0.15). Further, we found no effects of Sex (all *P* > 0.95) and no significant interaction between Age and Sex (all *P* > 0.27) for the number of wins, ties, or losses.

### Resident-intruder test

A GLM with Sex and Age as fixed factors yielded no effects or interactions for prosocial behavior, aggressive behavior, or nonsocial behavior (all *P* > 0.07) in the resident-intruder test. To examine the composition of behavior during the resident-intruder test, we compared the duration of test time spent exhibiting prosocial contact, aggressive behavior, and nonsocial behavior using Friedman’s test, which revealed a significant difference in the durations of different types of behavior that were exhibited (x^2^(2) = 82.55; *P* < 0.01). Posthoc analyses with Wilcoxon signed-rank tests showed that subjects overwhelmingly exhibited nonsocial behavior, but also exhibited more prosocial contact than aggression. All behavior types significantly differed from each other (all *P* < 0.01).

### Social approach

Each subject underwent 4 social approach tests with a novel opposite-sex conspecific, novel same-sex conspecific, familiar same-sex sibling/cagemate as stimulus animals, and a group of 3 novel same-sex conspecifics. We first used a RM GLM with Sex and Age as fixed factors and Stimulus Type as a within-subjects factor and found no effects or interactions for the latency to approach (all *P* > 0.09), time spent near the stimulus (all *P* > 0.39), or time spent investigating the stimulus (all *P* > 0.48). Because stimulus type did not influence behavior, we then ran LMMs with Sex and Age as fixed factors and Subject ID as a random factor to account for repeated testing to determine whether approach behaviors change with age or vary by sex regardless of social context. Sidak-corrected posthoc analyses revealed a main effect of Sex for the time spent investigating the stimulus animal (F_(1,79)_ = 4.301; *P* = 0.04), with males investigating stimuli more than females regardless of Age or Stimulus Type. However, we did not observe a main effect of Sex on the latency to approach or time spent near the stimulus animal (all *P* > 0.23). Further, we found no effects of Age (all *P* > 0.63) and no significant interaction between Age and Sex (all *P* > 0.06) for the latency to approach, time spent near, or time spent investigating the stimulus animal.

### Social interaction

**Fig 3:**
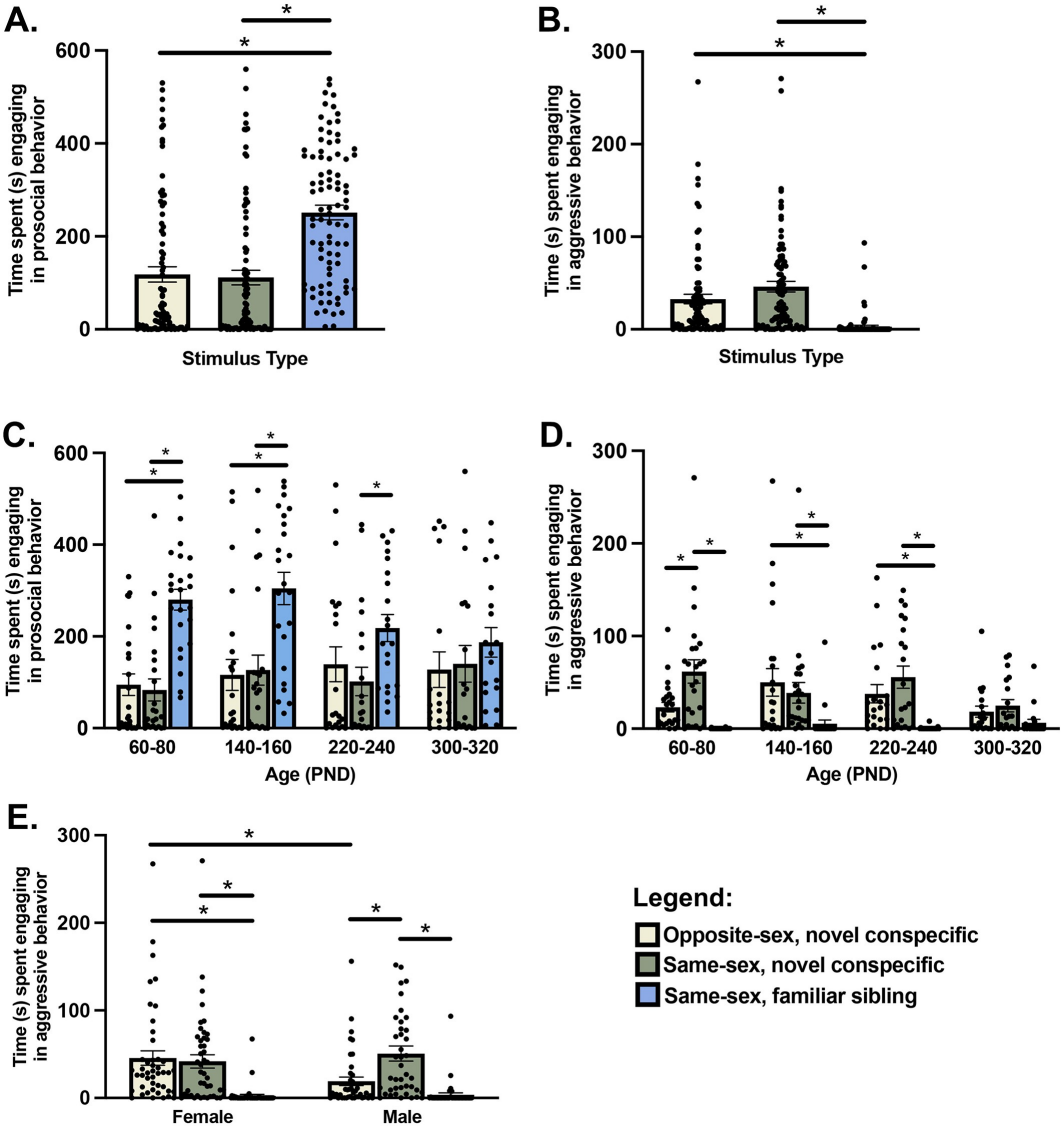
Mean ± SEM time (seconds, s) spent engaging in prosocial behavior and aggressive behavior in a social interaction test with a novel opposite-sex conspecific (beige), novel same-sex conspecific (green), and familiar same-sex sibling (blue). Male and female voles were **(A)** more prosocial and **(B)** less aggressive with their siblings compared to other stimulus types. **(C)** Prosocial behavior and **(D)** aggression varied by the type of conspecific voles interacted with for all age groups except the eldest group, PND300-320. **(C)** Aggression differentially varied by the type of conspecific males and females interacted with; females were more aggressive in interactions with opposite-sex conspecifics compared to males. Dots represent individual data points. * indicate statistical significance.

When examining prosocial behavior, a RM GLM with Sex and Age as fixed factors and Stimulus Type as a within-subjects factor yielded a main effect of Stimulus Type (F_(2,158)_ = 29.59; *P* < 0.01; Fig 3A). Sidak-corrected posthoc analyses revealed that subjects were significantly more prosocial with their familiar same-sex sibling/cagemate than the novel same-sex or opposite-sex conspecifics (both *P* < 0.01). We observed no effects of Sex or Age (both *P* > 0.49) and no interaction between Sex and Age on prosocial behavior (*P* = 0.15). However, we did find a significant interaction between Stimulus Type and Age for prosocial behavior (F_(6,158)_ = 2.167, *P* = 0.05; Fig 3C). Sidak-corrected posthoc analyses showed different patterns of prosocial behavior within age groups. Within age groups, animals aged PND60-80 and PND 140–160 were significantly more prosocial with their same-sex sibling than they were with either novel conspecific (both *P* < 0.01). Meanwhile, for animals in the PND220-240 age group, prosocial behavior did not differ when interacting with the novel opposite-sex conspecific and the same-sex sibling, but subjects were significantly more prosocial with their sibling compared to the novel same-sex conspecific (*P* = 0.01). However, for the eldest age group, PND300-320, prosocial behavior did not differ based on the type of stimulus animal the subjects interacted with (all *P* > 0.37). Further, within social context (i.e., Stimulus Type), posthoc analyses revealed no significant differences between age groups (all *P* > 0.07). Lastly, we did not observe interactions between Stimulus Type x Sex or Stimulus Type x Sex x Age (all *P* > 0.54).

When examining aggressive behavior, a RM GLM with Sex and Age as fixed factors and Stimulus Type as a within-subjects factor yielded a main effect of Stimulus Type (F_(2,158)_ = 27.11; *P* < 0.01; Fig 3B). Sidak-corrected posthoc analyses revealed that subjects were significantly less aggressive with their familiar same-sex sibling/cagemate than the novel same-sex or opposite-sex conspecifics (both *P* < 0.01). We observed no effects of Sex or Age (all *P* > 0.19). However, we observed a significant interaction between Stimulus Type and Age (F_(6,158)_ = 2.96; *P* < 0.01); Fig 3D). Sidak-corrected posthoc analyses showed different patterns of aggression within age groups, such that subjects aged PND60-80 were significantly more aggressive with novel same-sex conspecifics compared to novel opposite-sex or familiar sibling conspecifics (both *P* < 0.01). Conversely, age groups PND140-160 and PND220-240 exhibited a different pattern of context-specific aggression, with subjects being similarly aggressive with novel opposite-sex and same-sex conspecifics; animals in these age groups were significantly less aggressive with their same-sex sibling compared to the novel conspecifics (both *P* < 0.01). However, in the oldest age group, PND300-320, aggression did not differ across the 3 stimulus types (all *P* > 0.36). Within social context (i.e., Stimulus Type) we did not find any significant posthoc comparisons between age groups (all *P* > 0.07). Furthermore, we observed a significant interaction between Stimulus Type and Sex (F_(2,158)_ = 5.69; *P* < 0.01; Fig 3E). Sidak-corrected posthoc analyses found that for females, subjects were significantly less aggressive with their sibling compared to both the opposite- and same-sex novel conspecifics (both *P* < 0.01). However, for males, subjects were significantly more aggressive with novel same-sex conspecifics than with either the opposite-sex conspecific or their sibling (both *P* < 0.01). Additionally, when interacting with an opposite-sex conspecific, we found that females were more aggressive than males (*P* < 0.01). Lastly, we did not observe a higher order interaction between Stimulus Type x Sex x Age (*P* = 0.83).

When examining nonsocial behavior, a RM GLM with Sex and Age as fixed factors and Stimulus Type as a within-subjects factor yielded a main effect of Stimulus Type (F_(2,158)_ = 15.74; *P* < 0.01). Sidak-corrected posthoc analyses found that subjects were significantly less nonsocial with their sibling than with the novel opposite- or same-sex conspecifics (both *P* < 0.01). We observed no effects of Sex or Age (both P > 0.58) and no interactions including Sex or Age (all *P* > 0.13).

## Discussion

In the present study we examined the influence of aging on adult male and female prairie vole behavior. The age groups chosen for this study reflect the standard age range that researchers report for prairie vole study subjects. Whether PND320 can be considered “old” for a prairie vole remains unknown. In the wild, voles typically live less than 1 year of age, but death may be most likely due to predation. Further, there are currently no studies examining the progression of physical health in aging prairie voles, and thus we do not know when physical health starts to significantly deteriorate in voles. However, studies in mice show that physical function (i.e., locomotor activity, gait velocity, grip strength) begins to deteriorate around PND180, but cognitive functions (i.e., memory) do not exhibit impairment until roughly PND660 [41]. Therefore, our results should be considered within the context of behavior changing throughout vole adulthood, and caution should be taken to avoid categorizing the oldest age group in our study as “elderly” or “geriatric.” Here we found that the latency to approach a novel object, performance in a dominance test, and behavior in social interactions were significantly influenced by age. Perhaps the most striking finding was that prosocial behavior and aggression do not simply increase or decrease with age, but rather behavior changes with age relative to social context, and aged prairie voles cease to significantly alter their behavior based on social context. Here we discuss our findings in relation to advanced aging studies in other species as well as contextualize our results through consideration of prairie vole behavioral ecology.

### The impact of age on neophobia

Exhibiting caution to unfamiliar stimuli is common among many animals and likely serves as an adaptable survival mechanism [42, 43]. Novel object tests are frequently used to examine consistent individual differences in shyness-boldness, exploration-avoidance, or neophilia/neophobia [44]. Indeed, a recent meta-analysis found that novel object tests reliably quantify individual differences in behavior and yield a high repeatability of responses [45]. In the present study, we exposed male and female prairie voles to a novel object and examined the latency to approach the object, as well as the time spent near and investigating the object. We found that the youngest age group (PND60-80) exhibited the longest latency to approach a novel object, but only significantly compared to animals aged PND220-240. Additionally, animals in the youngest age group also yielded the greatest number of subjects (n = 10) that failed to approach the novel object before the test was terminated (see Fig 1). This finding suggests that young adult male and female prairie voles may be more neophobic than their older-aged peers. One can speculate that perhaps with age comes more experience and boldness. However, our findings stand in contrast to those of studies conducted in mice and rats. For example, a study in rats found that while weanlings do not exhibit neophobia toward novel food, young adults (PND92-170) and old-age adults (PND680-850) do exhibit neophobic responses [46]. Similarly, using a battery of tests to assess anxiety (i.e., novel object, exploration of novel environment, light/dark box, elevated plus maze), aging was associated with increased anxiety-like behavior in rats [47]. Consistent with this, another study found that middle-aged C57BL/6J mice (i.e., ~PND250-365) exhibit increased anxiety-like behavior compared to mice in young adulthood (i.e., PND60-90) in a light/dark box test [48]. Although the results in mice and rats do not align with ours, they do show that neophobia can be dependent on age. Theoretical models exist for both increases and decreases of neophobia with age; neophobia may decrease with age in some species as threats become less dangerous, whereas neophobia may increase with age in other species because they have more assets to protect [49, 50]. We had hypothesized that we would observe decreased neophobic behavior in young adult prairie voles, potentially serving to facilitate a phenotype conducive to exploring novel environments to find territories and/or mates. However, our novel object test results do not support this hypothesis and instead suggest that prairie voles may become less neophobic throughout adulthood. This is consistent with previous findings in aged male prairie voles, such that anxiety-like behavior in an open field test decreased with age [29]. Alternatively, it is feasible that behavior in the novel object test did not reflect anxiety-like behavior; rather, the nonsocial stimulus may have simply not been sufficiently interesting to be worthy of exploration, in which case younger voles may require more dynamic, salient stimuli to be motivated to readily approach and investigate something novel.

Although younger subjects were more cautious than older individuals in the presence of a novel object, we observed no effects of age on social approach behavior. Therefore, while it may be in the best interest of a young adult prairie vole to exhibit caution with novel *nonsocial* stimuli, potentially until they become more experienced, given the importance of finding a mate and establishing a pair bond to fitness [51], it may be critical to not exhibit *social* neophobia early on in adulthood. Our results support this possible interpretation and show that social neophobia is not influenced by adult age in prairie vole males or females. Interestingly, the type of stimulus also did not influence approach behavior in our animals. We were somewhat surprised that subjects approached the small group of novel, same-sex conspecifics at the same rate as other stimulus types. The small group was intended to [superficially] mimic the potential of encountering a novel prairie vole family while foraging or exploring outside the nest. Because prairie voles are territorial [52], we expected subjects to avoid a novel group, however, we found no differences in approach or investigative behavior based on stimulus type, suggesting that a novel, small group of same-sex conspecifics may not induce social neophobia in prairie voles. Field studies have shown that prairie voles have considerable overlap in home range territories [53] and when territorial vacancies arise, at least females, rapidly immigrate to occupy said vacancies [54]. Therefore, exhibiting a lack of social neophobia as an adult, at any age, may not only be crucial for finding mates, but also enables success in finding suitable territories.

Despite a lack of age effect on social approach behavior, we did observe an effect of sex on investigative behavior in the social approach test such that, regardless of age or social context, males investigated stimuli more than females. Consistent with this, recent studies demonstrated that while female prairie voles work harder to access a familiar mate over a novel, opposite-sex conspecific or a familiar conspecific over a novel conspecific, males work equally hard to access any kind of stimuli [55, 56]. Together, these findings suggest that male prairie voles are socially motivated by a more diverse range of social stimuli compared to females. Male prairie voles exhibit multiple mating strategies in the field, including a “resident” pairing strategy (note that paired males still exhibit extra-pair copulations) and a “wanderer” non-monogamous strategy [57], and thus investigating conspecifics regardless of novelty may prove more beneficial to males than females. Additionally, higher investigation rates in males may also reflect that males experience fewer consequences of interactions with opposite-sex individuals compared to females given that males do not give birth to and cannot nurse pups [58]. Therefore, it may be riskier for females to engage in social contact with novel conspecifics and therefore females exhibit less social investigation compared to males. This could have influenced our results and yielded a sex difference because social novelty was a factor in 3 out of 4 of our stimulus types in the social approach test. Future studies should directly test whether aspects of social neophobia are greater in female than male prairie voles regardless of age.

### The impact of age on territoriality

Here we found that dominance, as assessed by the tube test, varied by age, but only between two age groups, such that the eldest age group (PND300-320) exhibited significantly more losses compared to the second youngest age group (PND140-160). This could be a reflection of extremes in our sample such that PND140-160 aged voles may be closer to a stage of peak territoriality, at least compared to voles in advanced age, where losing competitions could result in infanticide of multiple litters of offspring, loss of a partner, or loss of a territory. Notably, the most losses were observed in the eldest age group and the greatest variation in the number of losses occurred within the two older age groups (see Fig 2), suggesting that dominance phenotypes may become less stable with age. Very few studies have used the dominance tube test in prairie voles. However, one study used this test to examine the relationship between dominance and alcohol intake and found that prairie voles aged PND75-95 exhibited consistent dominance phenotypes, suggesting the tube test has high efficacy in young adulthood [59]. Characterization of dominance phenotypes is more common in lab mice [60, 61]. Indeed, a recent study examined the effects of age on dominance in male ChAT-IRES-Cre mice using a social interaction test (e.g., number and duration of follow episodes, number of times the focal mouse placed paws on the head or back of the stimulus animal) and found that male mice aged PND60-90 were more dominant than PND335-365 males. Although our results, along with those found in mice, suggest that social dominance decreases with age, a study that tracked dominance over time in a free roaming group of 20 wild rats in an outdoor enclosure found that age, but not body weight, predicted dominance phenotype, with the older rats winning and maintaining higher ranks in the social hierarchy [62]. Whether these contrary findings in rats reflect a species difference or a methodological difference remains unknown. However, given that prairie voles live in small family groups, with many offspring dispersing to establish their own territories/families [63], it is likely that the parents may be the most dominant individuals in the group. Future studies are required to determine whether age and/or body weight influence dominance among cohabitating siblings and whether dominance phenotypes in the natal home are consistent during encounters with novel individuals outside the nest.

In the present study, we did not observe an effect of age on behavior in the modified resident-intruder test. Thus, in the context of a territorial intrusion, it may be characteristic of prairie voles to engage in aggression and defend their territory regardless of age. However, we observed that both male and female prairie voles in our study were overwhelmingly nonsocial during the modified resident-intruder test and primarily avoided the novel, same-sex conspecific intruder. Although some aggression was observed, low levels may reflect the short duration of our resident-intruder test; due to ethical considerations, Emory veterinary staff required a test no longer than 6 min to prevent potential injury. It is also possible that the lack of aggression may reflect the semi-neutral territory in which the subjects were tested. Although the subject was allowed to “establish residency” for 20 min in the test cage prior to the addition of the intruder, our subjects may have lacked sufficient motivation to defend their territory due to the recency of being placed in the novel cage. However, previous studies in prairie voles have shown that aggressive behavior does not differ during interactions with a novel same-sex conspecific in the homecage or a neutral cage [64]. Further, a recent study in which prairie voles were tested in a neutral cage for 10 min yielded high frequencies of aggressive behavior [52]. Thus, the low levels of aggression observed in our study may be the result of a shortened test period, and it is possible that we would have observed more aggression had the test been allowed to be carried out for longer. However, the composition of behavior exhibited in the resident-intruder assay in the present study does mirror our previous findings in male prairie voles aged PND30, 45, and 60, which also primarily exhibited nonsocial/avoidant behavior in a 10 min interaction with a novel same-sex conspecific [28]. Notably, when considering the duration of resident-intruder interactions in the wild, such interactions most likely typically last for fewer than 6 min. High levels of aggression in laboratory resident-intruder paradigms may be the result of being tested in a small cage with no opportunity to escape or leave. Therefore, our data may have some ethological-relevance and suggest that aggression and socially avoidant behavior in the context of a territorial intrusion does not differ with age in prairie voles.

### Adapting behavior to social context wanes with advanced age

In freely-behaving social interaction tests on neutral territory, we found that male and female prairie voles adjust their behavior accordingly dependent on social context (i.e., who they are interacting with). Not surprisingly, regardless of age or sex, subjects were significantly more prosocial and less aggressive with their familiar, same-sex siblings than they were with either the same- or opposite-sex novel conspecifics (see Fig 3). However, we found that altering behavior relative to social context waned with age. Subjects in the PND60-80 and PND140-160 age groups exhibited similar prosocial behavior profiles, such that they were more prosocial with their same-sex sibling/cagemate than with either the novel same- or opposite-sex conspecifics. Yet, for PND 220–240 animals, prosocial behavior did not significantly differ between the opposite-sex conspecific and the sibling, and for the eldest age group (PND300-320) prosocial behavior did not differ at all based on social context. Although it *appears* that prosocial behavior with novel conspecifics may have gradually increased with age and prosocial behavior with siblings may have decreased with age, we found no statistically significant interactions between Age and Stimulus Type, possibly due to the substantial amount of individual variation in our study. Rather, our data suggest that it is not as simple as ‘prairie voles become more or less prosocial with age,’ even within a specific context, but rather that prairie voles calibrate prosocial phenotype in a context-dependent manner in young adulthood and stop adjusting prosocial behavior to social context as they age. We find it somewhat perplexing that male and female prairie voles in young adulthood were not more prosocial with novel opposite-sex conspecifics given that finding a mate and pair bond partner should presumably be a high priority in young adulthood. However, despite our lack of an age effect on approach and investigative behavior in the social approach test, perhaps our results from the social interactions tests indicate that young adult prairie voles are indeed more socially neophobic than their elders. This would align with the object neophobia the youngest age group exhibited in the novel object task.

Similar to our findings for prosocial behavior, we also found that aggression profiles differed with age. PND60-80 prairie voles were more aggressive with a same-sex conspecific compared to a sibling and an opposite-sex conspecific. Although the exhibition of prosocial behavior did not differ in interactions with same- and opposite-sex conspecifics for PND60-80 individuals, it may be highly beneficial for this age group to at least suppress aggression when interacting with potential mates given that this age corresponds to the life history transition when prairie voles should be the most receptive to novel members of the opposite sex as they seek a partner to establish a pair bond and reproduce. Conversely, ages PND140-160 and 220–240 were similarly aggressive toward both same- and opposite- sex novel conspecifics. Even though prairie voles can still sire offspring at this age, it may be more likely that, in the wild, they would have already established a pair bond and therefore the need to suppress aggression with novel opposite-sex conspecifics may be waning. Further, our results suggest that in addition to exhibiting an onset of selective aggression with pairbonding [65], aggressive phenotype relative to social context changes in prairie voles with age regardless of pair bond status. Interestingly, though, the eldest age group (PND300-320) did not exhibit differences in aggression based on social context, suggesting that heightened aggression toward novel conspecifics dissipates with age. Taken together, our data do not support our original hypothesis that aggression generally decreases with age. In fact, we observed no effect of age (or Age *X* Stimulus Type interactions) on aggression in free-interactions during the social interaction tests or the resident-intruder tests. Instead, our data suggest that the selectivity of aggression changes with age, with male and female prairie voles becoming less selectively aggressive with advanced age.

Why might prairie voles stop altering their behavior in a context-specific manner as they age? One possibility is senility. Perhaps prairie voles lose the ability to discriminate between individuals in advanced age. Although no studies have specifically examined social discrimination in aging prairie voles, a previous study found that olfactory discrimination abilities are preserved in age and are relatively equivalent in 2-, 10-, and 23-month-old mice [66]. Indeed, olfactory discrimination abilities are crucial to survival in most rodents [67, 68], and it is likely highly adaptable to exhibit preserved sensory function throughout the lifespan of a rodent. Although somewhat different from our findings in the present study, two previous studies using C56BL/6J male mice found that PND180-365 mice engaged in less social contact in a social interaction test with a novel, same-sex juvenile compared to mice aged PND60-150 [48, 69]. Interestingly, reduced levels of social interaction preceded impaired performance in cognitive, sensory, and motor tasks, and the authors speculated that there may be a selective vulnerability of social circuits during aging [69]. This could suggest that cognitive, sensory, and motor functions may be spared for longer, possibly at the expense of social interest.

If prairie voles do not exhibit impaired social discrimination with age, then a waning expression of context-specific behavior could potentially be due to a decrease in social motivation. Perhaps social encounters with novel individuals are less important because in advanced age, prairie voles are less likely to form pairbonds and produce offspring. However, in labs, prairie vole breeders are typically used for extended periods of time, often beyond the ages that are chosen for subjects. One meta-analysis reported a maximum age at which a *first* litter was sired as PND422 for males and the birth of the *first* litter for mothers at a maximum age of 180 [70]. This demonstrates that prairie voles are still reproductively viable beyond the age of the subjects in the present study. Thus, it may not be specifically social motivation that declines with age in prairie voles, but instead general motivation. Supporting this, a study in transgenic mice showed that mice’s engagement in learning cost-benefit analysis declines with age, suggesting that mice undergo an age-related loss of motivation [71]. A similar study observed a steady age-related decrease in cognitive performance on a Choice Serial Reaction Time Task in mice (6-, 18-, and 24-months old), and that older mice still performed less well than younger mice even after controlling for task difficulty, again suggesting that aging is associated with changes in motivation [72]. These findings in mice, combined with our results that the eldest prairie voles exhibited the most losses in the dominance tube test and did not adjust social behavior to context in the social interaction tests, suggest that male and female prairie voles may experience a decline in motivation with age. However, further studies that explicitly test motivation are required to determine whether the lack of context-specific behavior in advanced age observed in the present study was due to an age-related decline in motivation rather than a decline in other cognitive capacities.

### Better with age? Prairie voles as a model for studying elderly social relationships

In humans, social relationships tend to improve with age such that older adults generally experience more satisfying and positive social relationships than younger adults [73]. Older adults engage in strategies to maximize positive experiences and actively avoid social conflict [74]. To overcome interpersonal problems, unlike young and middle-aged adults, older adults choose passive strategies, which have been deemed the most effective way to handle conflict [75, 76]. In the present study, one could interpret the waning expression of context-dependent social behavior in the eldest age group as prairie voles taking a more passive approach to social interactions in advanced age, which may be a more stable strategy for maintaining social connections while avoiding injury. As is with humans [77, 78], social companionship is crucial to the physical health of prairie voles [79, 80]. Further, unlike in middle-aged human adults, social activities not only increase positive affect but also decrease negative affect in elderly adults, suggesting that friends and social engagement may act as a buffer against the negative effects of aging [81]. Indeed, being socially engaged well into advanced age reduces the risk of heart disease, dementia, disability, and cognitive decline [78, 82, 83]. Prairie voles have been successfully used as a model for examining the stress buffering effects of bonding [15, 31, 84, 85]. Our data suggest that this line of research could be extended to use prairie voles as a model for studying the cognitive and physiological benefits of social relationships and social engagement in advanced age.

## Conclusions

Here we examined behavioral trajectories of aging male and female prairie voles and found that nonsocial neophobia, dominance, and context-dependent social behavior are influenced by adult age. Perhaps most strikingly, our findings suggest that aspects of social behavior (i.e., prosocial behavior and aggression) become less selective with advanced age in prairie voles. Whether the waning expression of context-dependent behavior with age is due to a decline in cognitive and/or sensory abilities or a decrease in social motivation remains to be studied. Regardless, our study demonstrates that social behavior is plastic not only throughout early development, but also well throughout adulthood. Notably, our study used virgin adult voles; given that the brain and behavior change with pairbonding in prairie voles [86–88], it is important to consider that behavioral changes throughout adulthood in sexually-experienced, pairbonded prairie voles may differ from those of virgin voles. The prairie vole community rightfully prides themselves in allowing for natural variation in studies, however, it may be worth considering statistically controlling for age of subjects, and potentially age of parents, as random factors or covariates in analyses. Additionally, given that labs use prairie voles as breeders well into advanced age, it is worth considering that parental behavior toward offspring and/or epigenetic consequences of aging parents may differentially influence offspring behavior and relevant underlying mechanisms. Together our study demonstrates that social behavior continues to change across the lifespan, and future studies investigating age-related changes in social neural circuitry may be of value in understanding the consequences of aging on social relationships.
